# Addressing racial and ethnic disparities in premature exits from permanent supportive housing among residents with substance use disorders

**DOI:** 10.1186/s12889-024-21169-2

**Published:** 2025-01-28

**Authors:** Talia J. Panadero, Sonya Gabrielian, Marissa J. Seamans, Lillian Gelberg, Jack Tsai, Taylor Harris

**Affiliations:** 1 Center for the Study of Healthcare Innovation, Implementation, and Policy (CSHIIP), Department of Veteran Affairs (VA) Greater Los Angeles, Los Angeles, CA USA; 2 Desert Pacific Mental Illness Research, Education, and Clinical Center (MIRECC), VA Greater Los Angeles, Los Angeles, CA USA; 3 Department of Epidemiology, UCLA Fielding School of Public Health, Los Angeles, CA USA; 4 Department of Psychiatry and Biobehavioral Sciences, UCLA David Geffen School of Medicine, Los Angeles, CA USA; 5 Department of Family Medicine, David Geffen School of Medicine at UCLA, Los Angeles, CA USA; 6 Department of Health Policy and Management, UCLA Fielding School of Public Health, Los Angeles, CA USA; 7 Office of Healthcare Transformation and Innovation, VA Greater Los Angeles Healthcare System, Los Angeles, CA USA; 8 Department of Veteran Affairs Central Office, National Center On Homelessness Among Veterans, Washington, DC USA; 9 School of Public Health, University of Texas Health Science Center at Houston, Houston, TX USA

**Keywords:** Permanent supportive housing, Substance use disorder, Homelessness, Health disparities

## Abstract

**Background:**

Permanent supportive housing (PSH) is an evidence-based practice for reducing homelessness that subsidizes permanent, independent housing and provides case management—including linkages to health services. Substance use disorders (SUDs) are common contributing factors towards premature, unwanted (“negative”) PSH exits; little is known about racial/ethnic differences in negative PSH exits among residents with SUDs. Within the nation’s largest PSH program at the Department of Veterans Affairs (VA), we examined relationships among SUDs and negative PSH exits (for up to five years post-PSH move-in) across racial/ethnic subgroups.

**Methods:**

We used VA administrative data to identify a cohort of homeless-experienced Veterans (HEVs) (*n* = 2,712) who were housed through VA Greater Los Angeles’ PSH program from 2016–2019. We analyzed negative PSH exits by HEVs with and without SUDs across racial/ethnic subgroups (i.e., African American/Black, Non-Hispanic White, Hispanic/Latino, and Other/Mixed [Asian, American Indian or Alaskan Native, and Native Hawaiian or Other Pacific Islander, and mixed race/ethnicity]) in controlled models and accounting for competing risk of death.

**Results:**

In competing risk models, HEVs with at least one SUD had 1.3 times the hazard of negative PSH exits compared to those without SUDs (95% CI: 1.00, 1.61). When stratifying by race/ethnicity, Other/Mixed race residents with at least one SUD had 6.4 times the hazard of negative PSH exits compared to their peers without SUDs (95% CI: 1.61–25.50). Hispanic/Latino residents with at least one SUD had 1.9 times the hazard compared to those without SUDs; however, this association was not statistically significant (95% CI: 0.85–4.37). African American/Black residents with at least one SUD had 1.2 times the hazard compared to those without SUDs (95% CI: 0.85–1.64), indicating no evidence of an association with negative PSH exits. Non-Hispanic White residents with at least one SUD had 1.1 times the hazard compared to those without SUDs (95% CI: 0.75–1.66), similarly indicating no evidence to suggest an association with negative PSH exits.

**Conclusions:**

These findings suggest relationships between SUDs and negative PSH exits differ between racial/ethnic groups and suggest there may be value in culturally specific tailoring and implementation of SUD services for these subgroups.

## Background

Permanent supportive housing (PSH), which combines subsidies for permanent and independent housing with field-based supportive services, is an evidence-based practice that addresses homelessness and its profound associated health and social disparities. PSH has demonstrated success in retaining homeless-experienced residents for up to two years, including those with substance use disorders (SUDs) [1–3]; however, SUDs are also one of the most significant contributing factors towards premature, unwanted (“negative”) PSH exits (e.g., eviction) and returns to homelessness [4–6]. The prevalence of SUDs varies across racial/ethnic subgroups, with increased prevalence among PSH residents who self-identify as racial/ethnic minorities compared to Non-Hispanic White residents who have experienced homelessness [7]. As such, there is a need to identify subpopulations of homeless-experienced residents with heightened vulnerabilities towards negative PSH exits and to provide these groups with supports that enhance equity in housing stabilization interventions.

Developed in the early 1990s, PSH draws upon principles of “Housing First,” providing affordable, low-barrier housing options to individuals experiencing homelessness, and accompanied by linkages to medical and mental health services. PSH case management and other field-based supportive services are guided by a harm-reduction approach, and do not mandate SUD treatment and/or sobriety [8–10]. There is substantial evidence that PSH reduces homelessness and increases housing stability for residents with SUDs [1, 2]. However, despite the effectiveness of the PSH model for residents with SUDs, substance use remains one of the most significant contributing factors towards housing instability [11], including within PSH programs [4, 6].

In partnership with the Department of Housing and Urban Development (HUD), the Department of Veterans Affairs’ (VA) Supportive Housing (HUD-VASH) program is the nation’s largest PSH initiative and a useful setting to examine disparities in PSH outcomes and inform improvement efforts. SUDs are highly prevalent among homeless-experienced Veterans (HEVs), estimated to have 60–76% prevalence [12, 13] compared to 11–18% among the general Veteran population [14, 15]. Moreover, relative to the general Veteran population, HEVs have greater racial/ethnic diversity [16, 17] and diversity among Veterans is only projected to increase in coming years [18]. As such, there is a need to assess racial/ethnic disparities in PSH outcomes to inform tailored and targeted strategies for mitigating these disparities and to ensure the provision of equitable VA medical care and social services across subgroups of HEV residents.

Existing literature has identified disparities in SUD diagnoses among Veterans who self-identify as racial/ethnic minoritized groups, including the underdiagnosis of SUDs among Hispanic/Latino Veterans [19]. Existing literature has also identified higher odds of housing instability among Veterans who self-identify as racial/ethnic minoritized groups [17]. However, we know little about the relationships between SUDs and PSH outcomes across racial/ethnic subgroups of Veterans. An analysis of PSH outcomes from the first wave of HUD-VASH voucher administration found HUD-VASH to be more effective in improving housing retention outcomes among Non-Hispanic White HEVs with SUDs compared to African American/Black HEVs with SUDs; this analysis specifically noted that interactions between SUDs and race/ethnicity were deserving of future study [20]. The interactions among SUDs, race/ethnicity, and housing outcomes in this context remain understudied. To fill this gap, among a cohort of HEVs housed through HUD-VASH in Los Angeles, we used administrative data to examine the relationships between SUDs and negative PSH exits, overall and by race/ethnicity, for up to five years post-PSH entry.

## Methods

### Sample and procedures

We used VA administrative data (from the Corporate Data Warehouse, CDW) and VA’s homeless registry (the Homeless Operations Management and Evaluation System, HOMES) to identify a cohort of HEVs (*n* = 2,933) housed through HUD-VASH at VA Greater Los Angeles between 2016–2019. VA Greater Los Angeles’ HUD-VASH program is the largest of any VA facility in the nation. In addition to financial subsidies for permanent housing, HUD-VASH provides field-based case management that includes linkages to medical and behavioral health services within and outside VA, including SUD treatment. We retrospectively captured housing information up to five-years post HEVs’ move-in date to PSH (i.e., through December 31, 2021).

To identify our analytic sample, we abstracted residents’ HUD-VASH records from HOMES, including information from case managers about move-in dates, retention in PSH, and HUD-VASH exits, when applicable. Though some residents exit HUD-VASH for positive reasons (e.g., income increases typically attributed to employment or disability claim attainment, relocation to other permanent housing) most residents who exit HUD-VASH case management do so for negative reasons (e.g., eviction, incarceration, or returns to homelessness). From the 2,933 HEVs who moved into Los-Angeles-based HUD-VASH in 2016–2019, we used CDW to exclude persons with missing data on key variables of interest, including race/ethnicity (*n* = 177) and marital status (*n* = 18). Those with “Other” marked as their reason for PSH exit (*n* = 26) were also treated as missing. Our final analytic sample included 2,712 residents. We retrospectively captured time between each resident’s PSH move-in date and the event of interest (i.e., PSH exit), competing event (i.e., death), or administrative censor (i.e., end of study follow-up [December 31, 2021]).

These data were originally abstracted for a project examining smoking behavior and housing outcomes among this cohort of HEVs. All study procedures were reviewed and approved by Department of Veteran Affairs Greater Los Angeles’ Institutional Review Board as constituting quality improvement.

### Measures

#### Conceptual framework

**Fig. 1:**
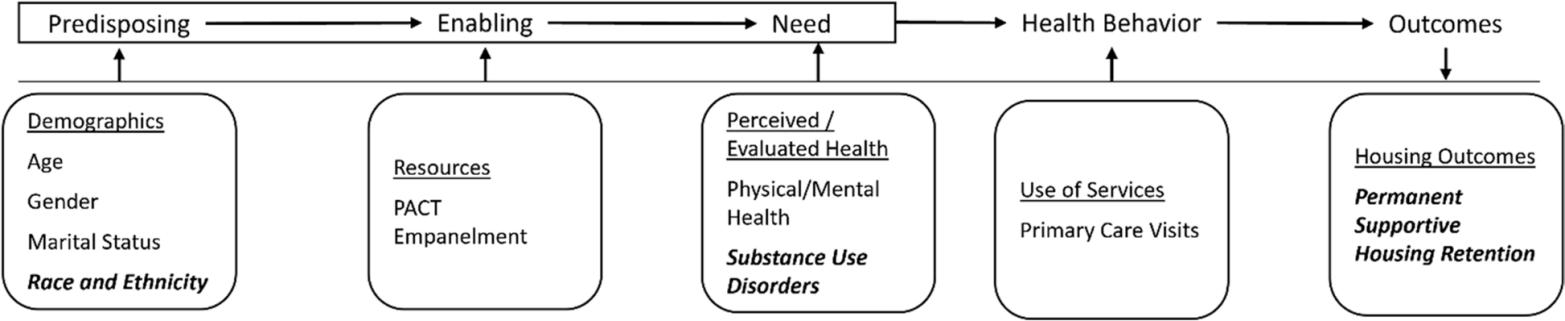
Conceptual framework adapted from the Behavioral Model for Vulnerable Populations (Gelberg, et al.) [ 21 ]

Measure selection and analyses were guided by the Behavioral Model for Vulnerable Populations [21] which describes person-level factors that predispose residents to health and housing outcomes (including age, gender, race/ethnicity, and marital status), which interact with characteristics that enable health access (e.g., primary care empanelment), needs (here, evaluated need for medical and mental health care), and health behaviors (e.g., primary care utilization) to influence HUD-VASH outcomes (retention or positive exits versus negative exits) (Fig. 1).

#### Predisposing factors

Demographic variables included *age* (modeled as a continuous variable at the time of move-in); *gender* (men and women); and *marital status* (stratified as married, previously married, or never married at the time of move-in) [11, 14].

For our key predisposing factor of interest, used to stratify the sample, we drew from VA administrative data which captures race across the following categories: African American or Black; White; Asian; American Indian or Alaskan Native (AIAN); Native Hawaiian or Other Pacific Islander (NHPI); Other Race; or Unknown. Ethnicity, a separate measure, captured Veterans identified as “Hispanic or Latino” versus “Not Hispanic or Latino”. To create a combined measure of *race and ethnicity*, we identified White and African American/Black patients who were not of Hispanic or Latino ethnicity, labelling these residents as “White, Non-Hispanic” (referred to as “White” hereafter) and “African American/Black, Non-Hispanic” (referred to as “African American/Black” hereafter), respectively. We collapsed residents of White race and Hispanic/Latino ethnicity into a “White, Hispanic/Latino” category (referred to as “Hispanic/Latino” hereafter). Residents who identified as a race other than White but with Hispanic ethnicity (e.g., African American/Black race and Hispanic/Latino ethnicity) were coded as “Other/Mixed race and ethnicity” (referred to as “Other/Mixed” hereafter). Asian, AIAN, and NHPI, and multi-racial Veterans were combined into the Other/Mixed category due to small sample sizes.

#### Enabling factors

We drew from the administrative data to determine Veterans empaneled to primary care, coined “Patient Aligned Care Teams” (PACTs), the VA’s patient-centered medical home model. We included Veterans assigned to specialty PACTS (e.g., Homeless-PACT [H-PACT] with providers and services tailored to HEVs) as empaneled. *Primary care empanelment* was modeled as a binary variable at the time of PSH move-in.

#### Need factors

Need factors were determined using diagnoses captured by primary or secondary International Classification of Disease, Tenth Revision (ICD-10) codes associated with VA outpatient or inpatient encounters in the administrative data over the two years prior to PSH move-in. ICD-10 codes associated with diagnoses are available in the supplemental materials.

Mental health diagnoses included in these analyses included schizophrenia and other psychotic disorders, bipolar disorders, post-traumatic stress disorder (PTSD), depressive disorders (e.g., major depression, dysthymia), and anxiety disorders (e.g., panic disorder, generalized anxiety disorder, social anxiety). Binary indicators for each mental health diagnosis reflect the presence of a visit for the given diagnosis versus the absence of a visit for the diagnosis. For mental health diagnoses, we modeled diagnoses separately due to their distinct relationships with housing retention as identified in prior literature [4, 22, 23]. Physical health diagnoses were ascertained via the Elixhauser Comorbidity Index Score [24], altered to exclude diagnoses already adjusted for in the study model (i.e., mental health diagnoses and substance use disorders).

For our key predictor variable of interest, we were focused on the presence or absence of *SUD diagnoses*, which we defined to encompass alcohol use disorder or any drug use disorder (including opioids, cannabis, sedatives/hypnotics or anxiolytics, cocaine, other stimulants, hallucinogens, inhalants, and other psychoactive substances).

#### Health Behaviors

Using administrative data, we characterized primary care utilization as the health behavior of interest. We captured primary care engagement in one-year post-PSH move-in, modeled as a binary variable (at least one primary care visit, yes or no).

#### Housing outcomes

Our outcome of interest was housing retention, which was captured through retention or exit from HUD-VASH PSH. Among residents who exited housing, their exit date was recorded along with a reason for exit in HOMES by case managers. We note that some residents in HUD-VASH exit rental units but remain enrolled in the program; we did not obtain that data, which is not available within VA’s homeless registry. Residents who were deemed to have negatively exited were confirmed by housing arrangement information (i.e., place not meant for habitation, transitional housing, shelter, treatment facility, or other temporary tenure), upon exit entered by case managers with the corresponding exit date. In the case of residents whose housing arrangement information was unknown, they were considered to have exited housing and presumed to have returned to homeless as the case manager could not locate them to determine their housing arrangement.

We stratified housing retention as: 1) retained (i.e., still housed at end of observation period; this included Veterans who exited the HUD-VASH program due to accomplishment of case management goals and/or no longer had need for case management and supportive services but remained housed; 2) positive or neutral exits; and 3) negative exits. We classified HUD-VASH exits as positive or neutral if they were associated with the following exit reasons: Veteran found/chose other housing; was no longer financially eligible for housing voucher (i.e., income was higher than eligible income rates); was escalated to a higher level of care; or was transferred to another HUD-VASH unit, e.g., in a different city or state. We classified negative exits as those attributed to other exit reasons, including: the Veteran cannot be located; did not comply with case management; was incarcerated; was no longer interested in participating in HUD-VASH; was unhappy with HUD-VASH housing; or was evicted and/or had other housing related issues or problems.

The outcome of interest was dichotomized (“Yes” or No”) as experienced a negative PSH exit versus the absence of a negative exit (i.e., a positive or neutral PSH exit or retained housing). Of note, we also used VA administrative data to identify residents who became deceased over the study period as opposed to exiting for other reasons, as this is a competing event (i.e., precludes the resident from exiting PSH during the study period).

#### Time-to-event

Each HEV was retrospectively followed beginning with their PSH move-in date and ending with either of the following events: the outcome of interest (i.e., PSH exit), a competing event (i.e., death), or the end of the study follow-up period (i.e., December 31, 2021)—whichever occurred first. We then calculated the time (in days) between each resident’s PSH move-in date and their respective event.

### Analyses

We characterized predisposing, enabling, and need factors among HEVs with and without SUDs. We did not compare exposed and unexposed groups or include inferential statistics (e.g., p-values) per the STROBE guidelines [25]. We retrospectively captured time between each resident’s PSH move-in date and the event of interest (i.e., PSH exit), competing event (i.e., death), or administrative censor (i.e., end of study follow-up [December 31, 2021]). Time-to-event (with PSH exit serving as “event”) data were used to calculate incidence rates. This was followed by survival analyses, using hazard functions, to compare occurrence of negative PSH exits among HEVs with SUDs versus those with no SUDs.

The proportional hazards assumption (i.e., that the relative hazards remain constant over time), which is the fundamental assumption for hazard regressions, was tested to examine if the effects of SUDs on negative PSH housing exits varied over time. In addition, we further tested the proportional hazards assumption to examine if the effects of SUDs on negative PSH housing exits varied over time within each racial/ethnic group. The proportional hazards assumption was not violated in any racial/ethnic group.

The reported incidence rates do not account for the competing risk of death. Therefore, to account for the competing risk of death in survival analyses, we fit Fine-Gray subdistribution hazard models, as this approach estimates hazards over time in the presence of competing events [26]. We estimated hazard ratios and 95% confidence intervals (95% CIs) for negative PSH exits comparing HEVs with SUDs to those without SUDs, accounting for the competing risk of death in multivariable models, and controlling for all other predisposing (age, gender, marital status), need (mental health diagnoses and Elixhauser score), enabling (primary care empanelment), and health behavior (primary care engagement) factors. These models were stratified across the four racial/ethnic subgroups to examine if the relationship between SUDs and negative PSH exits varied by racial/ethnicity. All analyses were conducted using Base SAS 9.4 ©.

## Results

### Sample characteristics

Table 1 describes the analytic sample (*n* = 2,712). Of the sample, 50% were African American/Black, 33% were White, 12% were Hispanic/Latino, 4% were Other/Mixed, and 40% had at least one SUD (Table 1). A majority (90%) of HEVs in the cohort were male and 88% were not married. The mean age at program entry was 53.4 ± 13.6 years. The average follow-up time (i.e., the average time between PSH move-in date and event of interest (i.e., PSH exit), competing event (i.e., death), or administrative censor (i.e., end of study follow-up [December 31, 2021]) was 3.0 years among HEVs with SUDs and 3.1 years among HEVs without SUDs. A minority (*n* = 397, 15%) of HEVs experienced a negative PSH exit*;* 225 (8%) died while in housing; most 2,090 (77%) were retained or experienced a positive/neutral PSH exit.

**Table 1:** Characteristics and negative permanent supportive housing (PSH) exits of HUD-VASH Veterans who entered PSH in 2016–2019 by having a substance use disorder (SUD) (*n* = 2712)

Sample Characteristics by Domains			Analytic Sample *N* = 2712		Analytic Sample by SUDs			
					*Substance Use Disorder* ***n* = 1077 (40%)**		*No Substance Use Disorder* ***n* = 1635 (60%)**	
Average follow-up time in years (mean ± sd)^a^			(3.1 ± 1.6)		(3.0 ± 1.6)		(3.1 ± 1.6)	
**Outcome**			**n**	**%**	**n**	**%**	**n**	**%**
PSH Retention or Positive Exit			2090	77	787	73	1303	80
Negative PSH Exit			397	15	183	17	214	13
Deceased			225	8	107	10	118	7
**Predisposing Factors**								
Gender								
Male			2436	90	1022	95	1414	867
Female			276	10	55	5	221	14
Age (mean ± sd)			(53.4 ± 13.6)		(53.8 ± 11.9)		(53.0 ± 14.5)	
Race and Ethnicity								
African American/Black, Non-Hispanic			1369	50	530	49	839	51
White, Non-Hispanic			908	33	371	34	537	33
White, Hispanic/Latino			316	12	145	13	171	10
Other/Mixed Race and Ethnicity^b^			119	4	31	3	88	5
Marital Status								
Married			333	12	106	10	227	14
Not Married			2379	88	971	91	1408	86
**Enabling Factors**								
PACT Empanelment			1,361	50	618	57	743	45
**Need Factors**								
Elixhauser^c^ (mean ± sd)			(2.1 ± 2.3)		(2.7 ± 2.4)		(1.8 ± 2.1)	
Mental Health Diagnoses								
PTSD			803	30	514	48	289	18
Schizophrenia			343	13	234	22	109	7
Bipolar			207	8	147	14	60	4
Depression			959	35	611	57	348	21
Anxiety			529	20	320	30	209	13
**Health Behavior**								
At least one primary care visit 1-year post PSH move-in			2025	75	925	86	1110	67

^a^Average time between PSH move-in date and event of interest (i.e., PSH exit), competing event (i.e., death), or administrative censor (i.e., end of study follow-up [December 31, 2021])

^b^Includes Asian, American Indian/Alaskan Native, Native Hawaiian/Pacific Islander, Other Race, and Multi-racial/ethnic

^c^Elixhauser Comorbidity Index Score is a measure of overall severity of comorbidities. The higher the score, the higher the comorbidities. In this analysis, the substance use and mental health diagnoses were removed from Elixhauser calculations to avoid over adjusting for SUDs and mental health diagnoses

Table 1 displays differences in needs and housing outcomes between HEVs with at least one SUD and HEVs without SUDs. HEVs with SUDs were more likely to be diagnosed with PTSD (48%), schizophrenia or other psychotic disorders (22%), bipolar disorders (14%), depressive disorders (57%), and anxiety disorders (30%) compared to HEVs without SUDs (18%; 7%; 4%; 21%; and 13% respectively). In addition, HEVs with SUDs had a higher mean number of physical health comorbidities compared to HEVs without SUDs (average Elixhauser score of 2.7 versus 1.8). HEVs with SUDs were also more likely to have at least one primary care visit within one year of PSH move-in date (86%) compared to HEVs without SUDs (67%). HEVs with SUDs also had a higher proportion of negative PSH exits (17% versus 13%) and a higher proportion of deaths (10% versus 7%), compared to those without SUDs.

## Associations between substance use disorders and negative PSH exits

The incidence of negative housing exits was slightly higher among HEVs with SUDs than in the group with no SUDs (incidence per 1,000 person-years = 56.6 vs. 42.2). HEVs with at least one SUD had 1.26 times the hazard of negative PSH exits compared to those without SUDs (cHR_Overall_ = 1.29; 95% CI = 1.06, 1.57). After controlling for predisposing, need, and enabling factors, the hazard ratio did not change materially (aHR_Overall_ = 1.27; 95% CI = 1.00, 1.61; see Table 2).

**Table 2:** Hazard ratio of negative permanent supportive housing (PSH) exits according to substance use disorder and race/ethnicity (*N* = 2712)

Race/Ethnicity	Substance Use Disorder	Total N	Total Person-Years	Negative PSH Exits^a^	Adjusted Hazard Ratio (95% CI)^b, c^
Total Complete-Case Population	No	1635	5077	42.2	*Reference*
	Yes	1077	3232	56.6	**1.27 (1.00, 1.61)**
African American / Black, Non-Hispanic	No	839	2659	43.6	*Reference*
	Yes	530	1659	54.2	**1.18 (0.85, 1.64)**
White, Non-Hispanic	No	537	1590	45.3	*Reference*
	Yes	371	1071	54.2	**1.12 (0.75, 1.66)**
White, Hispanic / Latino	No	171	534	31.8	*Reference*
	Yes	145	428	58.4	**1.92 (0.85, 4.37)**
Other / Mixed Race and Ethnicity^d^	No	88	295	30.5	*Reference*
	Yes	31	79	126.6	**6.41 (1.61, 25.50)**

^a^Per 1,000 Person-Years

^b^Using a Fine and Gray competing risk analysis

^c^Adjusting for gender, age, marital status, physical health diagnoses (Elixhauser Comorbidity Index Score), mental health diagnoses (PTSD, schizophrenia, bipolar, depression, and anxiety disorder)

^d^Includes Asian, AIAN, NHPI, Other Race, and Multi-Racial/Ethnic

## Stratification by race/ethnicity

**Fig. 2:**
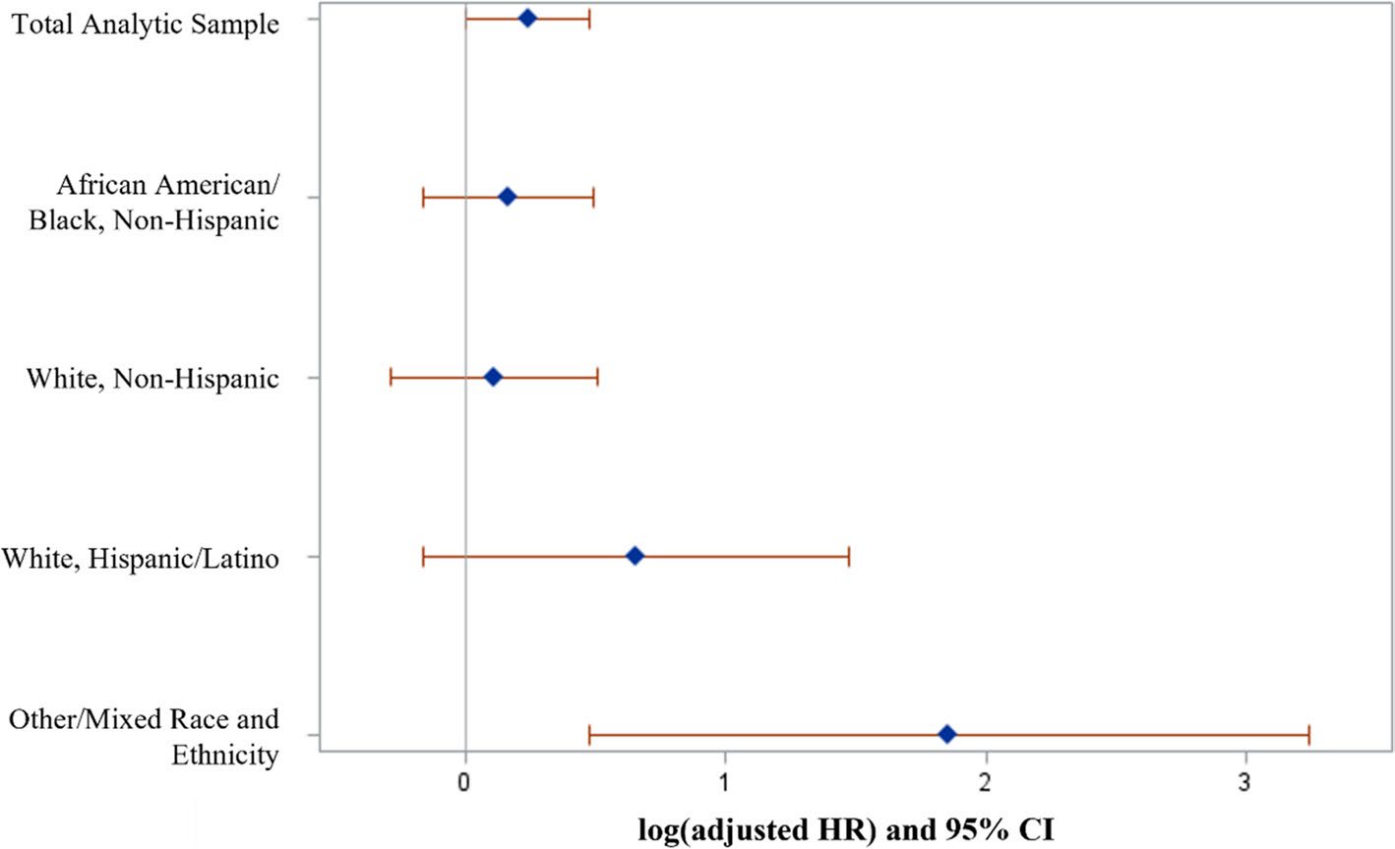
Effect of substance use disorder diagnoses on negative permanent supportive housing exits: log(adjusted hazard ratio) and 95% confidence intervals stratified by race/ethnicity

Table 2 also presents hazard ratios of negative PSH exits by SUD status, stratified by race/ethnicity. Other/Mixed race HEVs with at least one SUD had 6.4 times the risk of negative PSH exits compared to their peers without SUDs (aHR_Other/Mixed_ = 6.41, 95% CI: 1.61–25.50), whereas associations between SUDs and negative PSH exits were not statistically significant among African American/Black and White HEVs (aHR_Black_ = 1.18, 95% CI: 0.85–1.64; aHR_White_ = 1.12, 95% CI: 0.75–1.66) (Fig. 2). Hispanic/Latino HEVs with at least one SUD had 1.9 the hazard compared to those without SUD; however, this association was not statistically significant (aHR_Hisp/Latino_ = 1.92, 95% CI: 0.85–4.37).

## Discussion

We examined the relationships between SUDs and housing outcomes across racial/ethnic subgroups in a cohort of Veterans housed in HUD-VASH in Los Angeles. We identified an overall association between SUDs and negative PSH exits. However, in analyses stratified by race and ethnicity, we found this association varied by racial/ethnic group. There was no statistically significant association between SUDs and negative PSH exits for African American/Black, White, and Hispanic/Latino residents. Though it did not reach statistical significance, for residents of Hispanic/Latino ethnicity, the effect of presence of SUDs on negative PSH exits was nearly double that of White and African American/Black subgroups. We observed a statistically significant positive association for Other/Mixed race HEVs. Notably, the relationships between SUDs and negative PSH exits were much stronger among Other/Mixed HEVs compared to other racial/ethnic groups, although this group comprises a small subset of HEVs (4%).

Our findings differ from prior studies that broadly examined SUDs as associated with increased rates of premature or unwanted exits from PSH but did not focus on race/ethnic differences [6]. In these data, among most racial/ethnic subgroups, the effects of SUDs on negative PSH exits were not significant, which suggests that current strategies to retain residents with SUDs in PSH, (e.g., improving timely access to supportive services, including behavioral health care) may be effective among these subgroups [4]. Despite these efforts, however, our analyses highlight potentially important disparities in PSH housing outcomes among Hispanic/Latino and Other/Mixed race PSH residents with SUDs.

Among Hispanic/Latino and Other/Mixed race residents, disparities in health behaviors, including SUD service utilization, may contribute to the increased effect of SUDs on negative PSH exits. In prior literature, Veterans of Hispanic/Latino and Other/Mixed race and ethnicity were found to have SUD prevalence rates nearly two times that of clinically documented SUD [19]. Further indicating a gap in VA treatment receipt for SUD among these minoritized groups, White Veterans diagnosed with SUDs were found to be much more likely to receive treatment for SUD diagnoses as compared to Hispanic/Latino Veterans diagnosed with SUDs [27]. This trend is also seen among Asian and NHPI populations. Across the general population, outside of Veteran-specific literature, minoritized communities have been shown to severely underutilize SUD treatment. Underutilization among these populations is often attributed to barriers to access including stigma, cost, lack of knowledge, and cultural attitudes [28]. We suspect that tailored implementation approaches designed to increase adoption of evidence-based SUD treatments dissemination within VA (e.g., using peers to activate HEVs from racial/ethnic minoritized groups) may address these disparities and increase health equity within the PSH program.

Prior research has found that other potentially relevant factors in examining relationships between SUDs and negative PSH exits include socioeconomic disparities associated with developing SUDs [29], differential stigma associated with specific substance use [30], and other social factors associated with SUDs (e.g., disparate marketing for substances in low-income and minority communities [31]). Racial/ethnic minority Veterans are also noted to have an increased risk of adverse SUD and psychiatric treatment outcomes (e.g., involuntary hospitalizations, shorter treatment duration) compared to their Non-Hispanic White peers [32]. In general, researchers have attributed increased risk of SUDs among racial/ethnic minority populations to differential access to health services, social supports, and other healthy coping mechanisms (e.g., professional/clinic services, social service resources, community infrastructure). We note that, in this study, these disparities may be mitigated in part by the VA infrastructure; during the study period, all HUD-VASH residents were eligible for VA healthcare which awarded them equitable potential access to all health services, including SUD treatment.

## Strengths and limitations

The primary strength of this study is its ability to examine longitudinal data for a large subset of PSH enrollees in a system that integrates housing and health services. VA administrative and homeless registry data provides robust information related to diagnoses, date of housing move-in, exits from PSH enrollment, and the competing risk of death.

This study also had limitations. First, misclassification of PSH exits (i.e., negative, positive, neutral) may have occurred. Each exit is categorized using standardized reasons for exit which omit granular details about factors contributing to each participant’s PSH exit. Second, while there a large sample size for the entire cohort, when stratifying by race/ethnicity, small proportions in some subgroups (i.e., Asian, AIAN, NHPI, Other, and Mixed) necessitated collapsing of these subgroups into one category (“Other/Mixed”) which comprised 4% of HEVs. Given the diversity within the Other/Mixed category, as well as prior research pointing to SUD disparities among multiracial populations [33, 34], future studies would benefit from exploring these distinctions further. Future studies with larger samples sizes and/or utilizing qualitative methods could help provide greater insights into the potential vulnerabilities of racial/ethnic subgroups with smaller populations, including multiracial populations. Third, these analyses were based on diagnosed and documented SUDs, which may vary by race/ethnicity. In addition, in this study, we combined all diagnoses of substance use disorders within the relevant time frame (two years prior to housing move-in). Future research would benefit from examining differences in housing retention associated with specific substances used. We note specific complexities in data interpretation related to persons who only had cannabis use disorder to classify them as having a SUD; cannabis was legalized in the state of California in 2016, including at the study site [35]. Fourth, it is possible that the high rates of comorbid mental health disorders and SUDs among this population overshadowed effects of SUDs on negative PSH exits. Future studies may benefit from assessing the relationships between comorbid mental health and SUD diagnoses on housing outcomes. Fifth, additional characteristics not observed in the present study (e.g., socioeconomic status) may be associated with both race/ethnicity and SUDs, potentially helping explain the reported observed association between SUDs and PSH outcomes among certain racial/ethnic subgroups [36]. Last, as a study conducted with one large and urban VA, it is unclear how much our findings extrapolate to a national HUD-VASH sample, or to homeless-experienced consumers who receive PSH services or health services outside the VA.

## Conclusions

When stratifying by race and ethnicity, this study identified a strong association between SUDs and negative PSH exits among HEVs of Other/Mixed race and ethnicity compared to PSH residents of other race/ethnicity groups. These findings suggest that PSH programs and providers should consider potential heightened vulnerabilities for negative housing outcomes among minoritized residents, particularly those of Asian, NHPI, AIAN, and Other/Mixed race and ethnicity. These findings would benefit from integration with qualitative data that explores potential reasons for differential rates for negative exits among PSH residents of different race/ethnicity groups. Such research could inform culturally-specific tailoring of SUD services and implementation strategies that support equitable use of SUD services within these subgroups, which ultimately have potential to reduce Veteran homelessness and increase health equity.

## Data Availability

The datasets used and/or analyzed during the current study are available from the corresponding author on reasonable request. A Limited Dataset (LDS) will be created and shared pursuant to a Data Use Agreement (DUA) appropriately limiting use of the dataset and prohibiting the recipient from identifying or re-identifying (or taking steps to identify or re-identify) any individual whose data are included in the dataset.

## Abbreviations

AIAN: American Indian or Alaskan Native
CDW: Corporate Data Warehouse
CI: Confidence interval
HEV: Homeless-experienced Veteran
HOMES: Homeless Operations Management and Evaluation System
HR: Hazard ratio
HUD: Department of Housing and Development
ICD-10: International Classification of Disease, Tenth Revision
NHPI: Native Hawaiian or Other Pacific Islander (NHPI)
PACT: Patient Aligned Care Teams
PSH: Permanent supportive housing
sd: Standard deviation
SUD: Substance use disorder
VA: Department of Veteran Affairs
VASH: Department of Veterans Affairs’ Supportive Housing
